# Inhibitory receptor states, immune homeostasis and the benefit–risk boundary of cancer checkpoint blockade

**DOI:** 10.3389/fimmu.2026.1762484

**Published:** 2026-08-06

**Authors:** Xiaodong Wang, Wei Gao, Qianqian Wang, Songjiang He, Songli Cui, Bingchen Duan, Gouping Ding, Yiping Huang, Bingwen Zou

**Affiliations:** 1 Department of Radiation Oncology, Cancer Center and Institution of Stress Medicine, West China Hospital, Sichuan University, Chengdu, China; 2 The Center of Nuclear Stress Medicine, The Second Affiliated Hospital (Nuclear Industry 416 Hospital), Chengdu Medical College, Chengdu, China; 3 Department of Oncology, Zhuzhou Hospital Affiliated to Xiangya School of Medicine, Central South University, Zhuzhou, China; 4 Department of Orthopaedic Surgery, Zhuzhou Hospital Affiliated to Xiangya School of Medicine, Central South University, Zhuzhou, China

**Keywords:** benefit–risk stratification, immune checkpoint blockade, immune homeostasis, immune-related adverse events, inhibitory receptors

## Abstract

Immune checkpoint blockade has transformed cancer treatment, but its clinical success depends on releasing antitumor immunity without collapsing peripheral tolerance. Biomarkers such as PD-L1 expression, mismatch-repair deficiency and tumor mutational burden capture only selected aspects of this balance and provide limited guidance on immune-related adverse events (irAEs). This Review examines inhibitory receptor states as dynamic readouts of the therapeutic window in cancer immunotherapy. Rather than treating PD-1, CTLA-4, LAG-3, TIM-3, TIGIT, VISTA and NKG2A as isolated abundance markers, we discuss how their signaling intersects with CD3, CD28 and cytokine-driven activation, immune-cell differentiation, tissue localization and homeostatic restraint. We further consider how checkpoint blockade converts these circuits into either tumor control or organ-specific immune injury. Evidence from tissue pathology, blood immunomonitoring, single-cell and spatial multi-omics, routine laboratory markers, imaging and emerging host-immune surrogates is integrated into a longitudinal, regimen-specific framework. We argue that clinically useful models should jointly estimate response and toxicity risk, distinguishing reinvigoratable antitumor states from fixed dysfunction and autoreactive vulnerability. Such an approach could support patient selection, regimen tailoring, early toxicity surveillance and more precise use of combination immunotherapy.

## 1 Introduction

Immune checkpoint blockade has fundamentally altered the natural history of several cancers. Durable regressions and prolonged survival are now attainable in malignancies such as melanoma, non-small cell lung cancer, and renal cell carcinoma, conditions previously defined by rapid therapeutic failure (1–3). Yet these treatments remain ineffective for a substantial fraction of patients, and the clinical burden of immune activation is often considerable. Across multiple indications, objective response rates to PD-1 or PD-L1 blockade in unselected populations typically range from 15% to 40%, with only a minority of patients achieving durable long-term survival or progression-free intervals exceeding two to four years (1, 3, 4), whereas immune-related adverse events can involve virtually any organ system and may be severe, chronic, or fatal (5, 6).

The principal challenge is therefore no longer merely to unleash antitumor immunity, but to do so with sufficient biological precision to maintain a viable therapeutic window.

Current biomarker strategies, including tumor PD-L1 expression, mismatch-repair deficiency, and tumor mutational burden, enrich for clinical benefit in selected settings yet remain neither universally necessary nor sufficient, as evidenced by responses in biomarker-negative patients and non-responses in biomarker-positive cohorts across pivotal trials (7–9). These markers assess tumor immunogenicity or target expression in an indirect and fragmentary manner. They are also susceptible to intratumoral heterogeneity, assay discordance, temporal changes during treatment, and the inherent mismatch between a single pretreatment biopsy and a therapy that rapidly remodels immune states (8, 10, 11). Most importantly, the biomarkers currently approved for routine use were developed to predict efficacy rather than toxicity and thus provide scant information on the likelihood that treatment will disrupt self-tolerance (2, 4).

Inhibitory receptor profiling is attractive precisely because it interrogates the pharmacologic substrate of checkpoint blockade more directly. PD-1, CTLA-4, LAG-3, TIM-3, TIGIT, VISTA, and NKG2A are not merely therapeutic targets; they function as dynamic reporters of immune history (12, 13). Their expression encodes information about antigen exposure, duration of stimulation, lineage commitment, metabolic adaptation, and the requirement for physiologic restraint. Measured in tumor tissue, these receptors can indicate whether a lesion contains tumor-reactive lymphocytes poised for reinvigoration, bystander cells with limited relevance to tumor control, suppressive regulatory populations, or myeloid barriers resistant to current therapies. Measured in blood, they afford a serial view of pharmacodynamic remodeling that may precede radiographic response or clinical toxicity (12–15).

Interpretation of inhibitory receptor expression as simple abundance metrics frequently overstates their predictive value, because the functional significance of PD-1 or LAG-3 upregulation is contingent upon co-expressed activating signals, the differentiation state of the expressing cell (stem-like progenitor versus terminally exhausted effector), and the tissue microenvironment (12, 13). A coherent framework must therefore integrate inhibitory receptor signaling with the activating systems they restrain, the homeostatic programs they preserve under physiological conditions, and the tissue-specific circuits that fail during immune-related toxicity (5, 13, 16).

The present review develops this framework and argues that the most clinically useful application of inhibitory receptor profiling is not binary patient selection for a single agent, but rather the joint estimation of benefit and harm within a regimen-specific, longitudinal model.

## 2 Inhibitory receptor biology in immune homeostasis and checkpoint pharmacology

### 2.1 Signal integration rather than receptor abundance alone

Many biomarker efforts in cancer immunotherapy have rested on the premise that inhibitory receptor abundance alone (e.g., PD-1 or PD-L1) reliably identifies therapeutically actionable states, yet this view overlooks their embedding within integrated signaling circuits whose output depends on cellular context (13, 17, 18). Inhibitory receptors instead function as nodes embedded within larger signal-integration circuits. PD-1, for example, recruits phosphatases that blunt proximal signaling events downstream of the T-cell receptor and CD28, thereby suppressing PI3K-AKT-mTOR activity, glycolytic metabolism, proliferation, and cytokine secretion (13, 17). CTLA-4 operates through a different route: it raises the threshold for T-cell priming by competing with CD28 for CD80 and CD86 ligands on antigen-presenting cells and by actively removing these ligands through transendocytosis (19, 20). LAG-3 limits T-cell function through peptide-MHC class II and FGL1, while TIM-3 and TIGIT engage distinct ligands (CEACAM1, phosphatidylserine, CD155/CD112) that reinforce exhaustion or regulatory programs distinct from PD-1 (21, 22). VISTA and NKG2A extend comparable regulatory logic into myeloid and natural killer cell compartments (23, 24).

These pathways evolved to maintain immune homeostasis. Under physiological conditions they calibrate the strength, duration, and anatomical reach of immune activation, protecting tissue integrity amid infection, cell turnover, and commensal exposure. Their expression therefore frequently signals successful containment of immune damage rather than any intrinsic failure of immunity. High checkpoint levels on a chronically stimulated tumor-reactive T cell can mark retained antigen experience and pharmacological accessibility, creating an opportunity for therapeutic reinvigoration (12, 13, 18). By contrast, the same receptors expressed on regulatory T cells or tissue-resident populations often mark active homeostatic restraints, whose pharmacologic release can increase the risk of organ-specific immune injury (16, 25). Consequently, within one patient the same receptor measurement can simultaneously indicate potential benefit and potential harm.

This contextual dependence is decisive when receptor profiles are used for prediction. A tumor enriched for PD-1^high CD8 T cells may contain reinvigoratable antitumor clones, yet it may equally contain cells whose dysfunction has become fixed through epigenetic remodeling and is therefore resistant to rescue (13, 15, 18). An expanded checkpoint-positive population in blood may represent a poised antitumor reserve, a state of broader systemic activation, or a compensatory response to emerging tissue pathology (14, 26). Receptor abundance therefore carries information only when read together with differentiation state, concurrent activating signals, and temporal kinetics (13, 18).

### 2.2 Cell lineage, differentiation, and spatial context

Checkpoint molecules exert lineage-specific functions that differentially influence antitumor efficacy and toxicity risk (13, 24). Within CD8 T cells, multi-receptor expression is commonly interpreted through the lens of exhaustion, yet exhausted populations exist along a continuum rather than as a uniform state (15, 18). Stem-like or progenitor-exhausted cells typically retain TCF1, preserve proliferative capacity, and remain responsive to PD-1 blockade (12, 27). Terminally exhausted cells co-express multiple inhibitory receptors, display strong but transient cytotoxicity, and show limited persistence (18). Tumors that retain substantial progenitor pools are more likely to achieve durable benefit after checkpoint blockade; tumors dominated by terminal states may exhibit brisk initial responses that prove incomplete or short-lived (12, 18).

In CD4 T cells, functional implications differ markedly: PD-1 is frequently expressed by follicular helper T cells and cells within tertiary lymphoid structures in organized immunity, whereas tumor-infiltrating Tregs commonly co-express CTLA-4, TIGIT and LAG-3 to enforce suppression (16, 25, 28). An intervention that restores effector function in exhausted CD8 T cells can simultaneously destabilize these regulatory compartments, eroding dominant tolerance and contributing directly to immune-related adverse events (16, 29).

Natural killer cells and myeloid populations introduce additional layers. NKG2A restrains cytotoxicity in NK cells and selected CD8 T-cell subsets, particularly in tumors that exploit HLA-E to evade both innate and adaptive killing (24, 30). VISTA, by contrast, is enriched in myeloid-rich environments and frequently identifies tumors in which suppressive macrophages and myeloid-derived suppressor cells predominate over lymphoid inflammation. In such lesions the principal barrier to therapy may reside in myeloid architecture rather than classical T-cell exhaustion, rendering PD-1 blockade alone insufficient (21, 23, 31).

**Figure 1:**
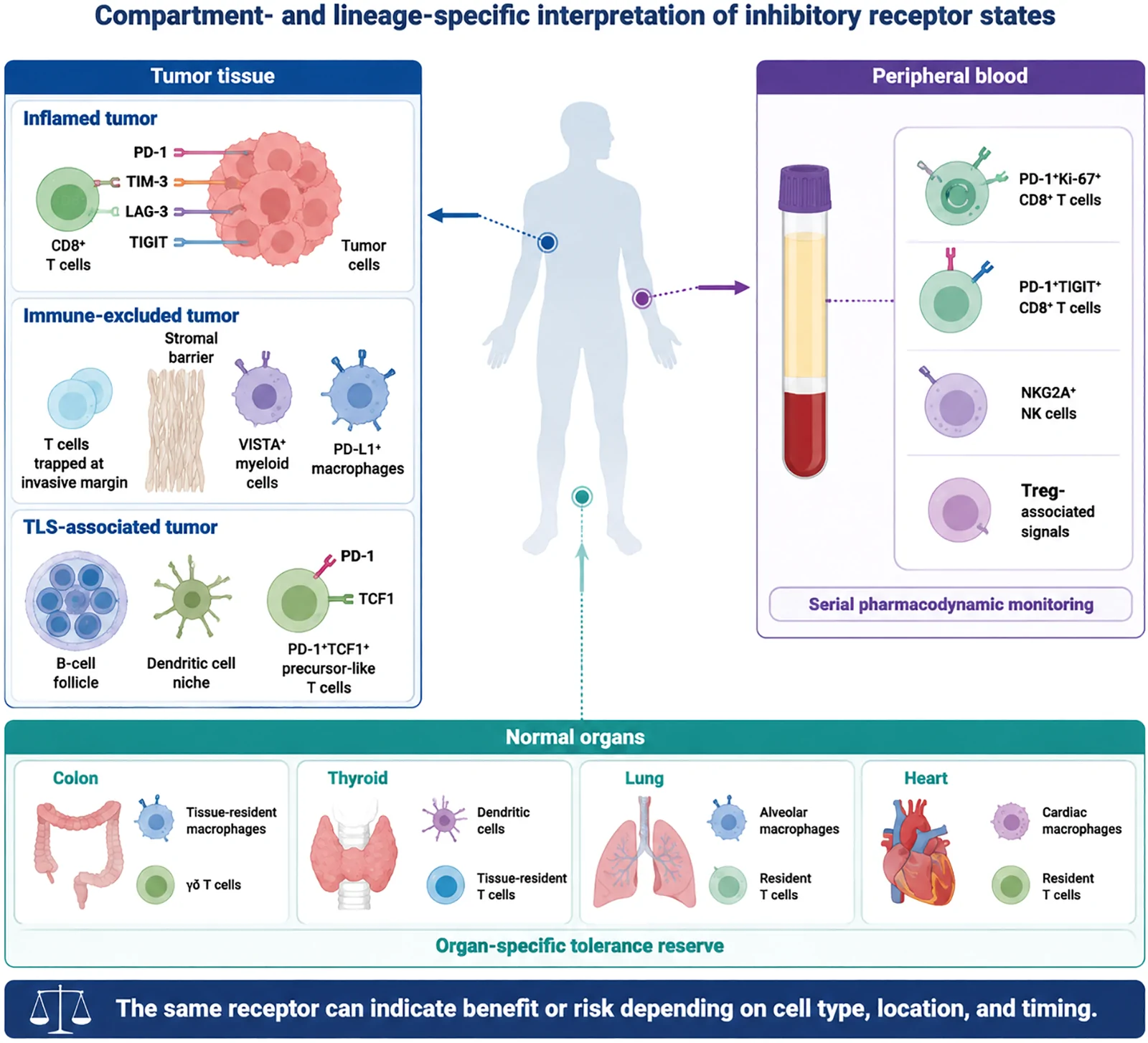
Compartment- and lineage-specific interpretation of inhibitory receptor states. In tumor tissue, receptor expression acquires different meanings in inflamed, immune-excluded and tertiary lymphoid structure-associated contexts, ranging from targetable PD-1+ CD8+ T-cell states to VISTA+ myeloid barriers and PD-L1+ macrophage-rich exclusion. In peripheral blood, serial measurements capture pharmacodynamic changes in PD-1+Ki-67+ CD8+ T cells, PD-1+TIGIT+ CD8+ T cells, NKG2A+ NK cells and regulatory T-cell-associated signals. Normal organs add a third interpretive layer, because colon, thyroid, lung and heart each contain tissue-resident immune populations that define organ-specific tolerance reserve.

Anatomical location within tissue further determines biological significance. Spatial analyses reveal that checkpoint-positive cells situated at the invasive margin, within tertiary lymphoid structures, in stromal exclusion zones, or in the tumor core can exert markedly different effects on tumor control and therapeutic outcome (28, 32, 33). Not every PD-1-positive tumor-infiltrating T cell represents a tumor-reactive clonotype; many are bystander cells with minimal relevance to tumor eradication (32, 34). Peripheral-blood measurements likewise cannot be assumed to mirror the tumor microenvironment, although serial sampling may detect early pharmacodynamic signs of reinvigoration that a single pretreatment biopsy cannot capture (14, 26, 35). Any translational use of inhibitory receptor profiling must therefore remain compartment-aware (**Figure 1**).

### 2.3 Activating receptor systems as the counterweight to inhibitory circuits

Inhibitory receptor pathways do not operate in isolation; their functional impact is determined by dynamic integration with activating signals from antigen receptors, co-stimulatory molecules and cytokines, such that the biologically relevant unit is the balance between inhibitory tone and activating drive (13, 17, 19). On T cells, activating drive is generated through antigen-receptor signaling via proximal CD3-associated pathways, CD28 co-stimulation, and cytokine networks (including IL-2, IL-7, IL-15 and type I interferons) that collectively support survival, proliferation and differentiation (17, 36). PD-1 is especially effective because it attenuates both T-cell receptor output and CD28-dependent signaling, making net cellular behavior the integrated product of brake and throttle (17, 26, 36). CTLA-4 exerts its major influence during priming by intercepting the CD80 and CD86 ligands required for CD28 engagement (19, 37).

**Figure 2:**
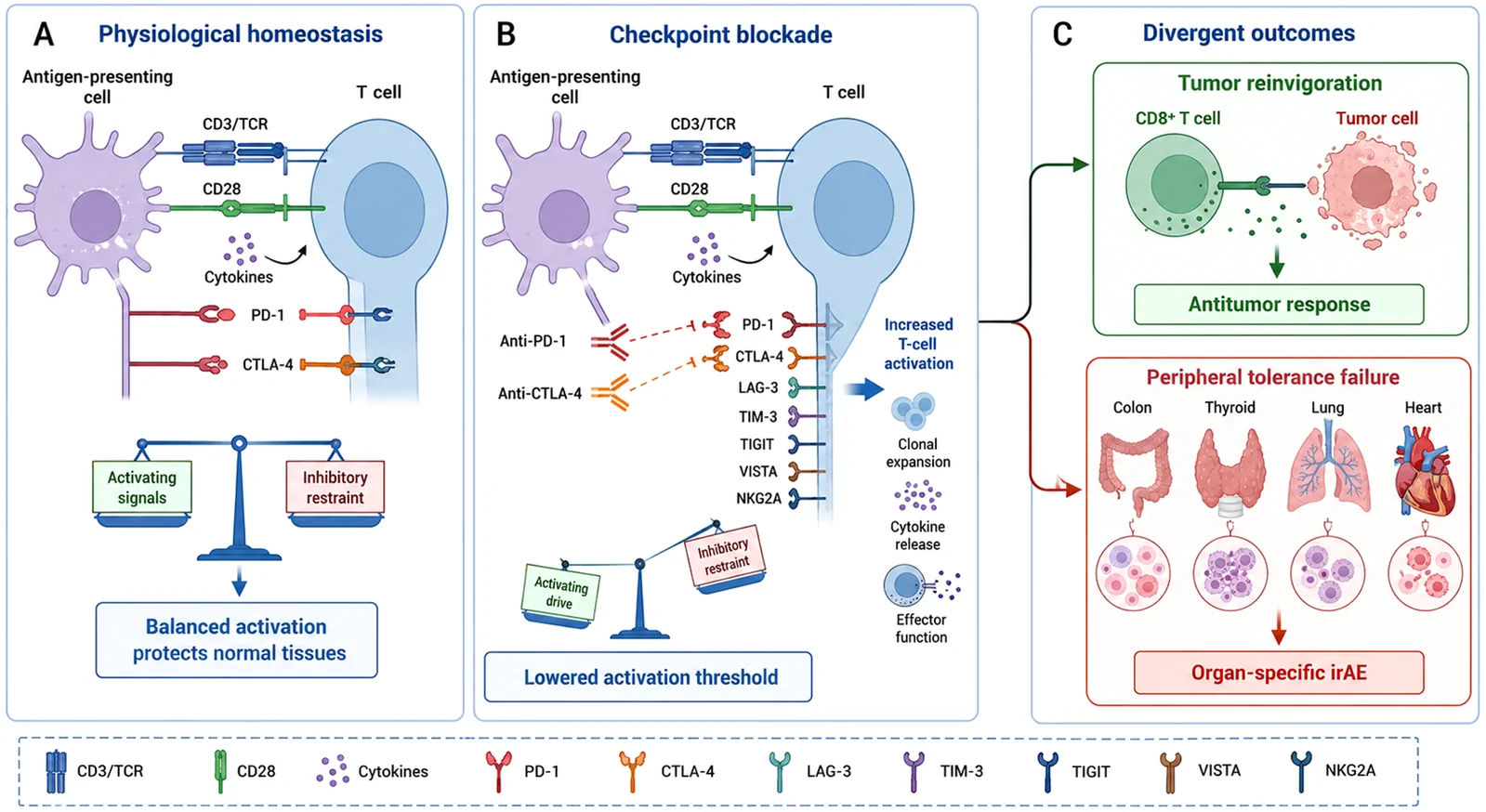
Signal integration links checkpoint blockade to either tumor reinvigoration or peripheral tolerance failure. **(A)** Under physiological conditions, CD3/TCR, CD28 and cytokine signals are counterbalanced by inhibitory receptors, maintaining controlled T-cell activation and protecting normal tissues. **(B)** PD-1 or CTLA-4 blockade lowers the activation threshold and promotes clonal expansion, cytokine release and effector function, while additional checkpoints such as LAG-3, TIM-3, TIGIT, VISTA and NKG2A shape residual restraint. **(C)** The same pharmacological release can produce divergent outcomes: productive CD8+ T-cell-mediated tumor control or organ-specific immune-related adverse events involving colon, thyroid, lung and heart.

Several practical consequences follow. First, high inhibitory receptor expression in the absence of concurrent activation may indicate immune quiescence rather than therapeutically actionable exhaustion; “checkpoint-high” does not automatically mean “checkpoint-dependent.” Second, the sufficiency of activating receptor systems helps distinguish productive inflammatory states from fixed dysfunction. Stem-like PD-1-positive T cells that retain CD28-linked responsiveness, proliferative reserve, and clonal plasticity are far more likely to expand productively after blockade than terminal populations marked by metabolic insufficiency and durable chromatin remodeling (12, 13, 15). Third, activating cues are indispensable for toxicity: loss of inhibitory restraint becomes clinically dangerous only when concomitant activating signals are strong enough to propagate effector function into normal tissues (**Figure 2**) (16, 29, 38).

These activating cues arise from sources beyond tumor antigen. Cytokines including IL-2, IL-7, IL-15, type I interferons and IFN-γ sustain lymphocyte survival, trafficking and effector differentiation while activating myeloid cells; their presence can thereby potentiate both antitumor reinvigoration and tissue inflammation following checkpoint blockade (16, 29, 38). In chronic inflammatory settings, even modest checkpoint disruption can escalate into widespread tissue injury. B-cell activation and autoantibody production have been observed in association with selected immune-related adverse events and may contribute to tissue pathology in susceptible individuals through loss of peripheral tolerance (39–41). A coherent biomarker model must therefore treat inhibitory receptor profiles as one coordinate within a multidimensional immune state, to be interpreted alongside co-stimulatory competence, inflammatory cytokine tone, and innate activation. A receptor signature considered apart from its activating context is incomplete by design (**Table 1**).

**Table 1:** Mechanistic interpretation of inhibitory receptor states across immune homeostasis, antitumor reinvigoration, and immune-related toxicity.

Receptor or signaling axis	Principal cellular context	Homeostatic function	Interpretation for efficacy prediction	Interpretation for irAE prediction	Reference
PD-1–CD3/CD28–PI3K–AKT–mTOR axis	Activated, chronically stimulated, and exhausted CD8^+^ and CD4^+^ T cells	Dampens TCR and CD28-dependent signaling, restrains metabolic reprogramming, proliferation, and effector cytokine production	Moderate PD-1 expression, particularly in proliferative or precursor-like CD8^+^ T-cell states, may indicate targetable antitumor immunity capable of reinvigoration after PD-1 blockade	Loss of PD-1 restraint may expand autoreactive or tissue-resident effector populations, particularly when accompanied by strong inflammatory drive	Patsoukis et al. (36), *Nature Communications*, 2015; Kamphorst et al. (26), *PNAS*, 2017.
CTLA-4–CD80/CD86–CD28 axis	Naïve and recently primed T cells, regulatory T cells, lymphoid priming sites	Raises the threshold for T-cell priming by competing with CD28 and removing CD80/CD86 from antigen-presenting cells	CTLA-4^high regulatory or priming-associated states may identify tumors requiring broader immune activation beyond PD-1 monotherapy	CTLA-4 blockade can destabilize regulatory tolerance and is therefore strongly linked to systemic and gastrointestinal irAEs, especially in combination regimens	Walunas et al. (20), *Immunity*, 1994; Kennedy et al. (19), *Nature Immunology*, 2022.
LAG-3–MHC-II/FGL1 axis	Exhausted CD8^+^ T cells, regulatory T cells, selected CD4^+^ subsets	Restricts effector T-cell function and reinforces regulatory or exhaustion programs	PD-1^+^LAG-3^+^ exhausted T-cell enrichment may support use of dual PD-1/LAG-3 blockade when PD-1 monotherapy is biologically insufficient	Dual release of PD-1 and LAG-3 may intensify effector activation; toxicity risk should be interpreted together with systemic activation and host susceptibility	Wang et al. (21), *Cell*, 2019; Maruhashi et al. (22), *Immunity*, 2022; Andrews et al. (31), *Cell*, 2024.
TIM-3 and TIGIT pathways	Terminally exhausted CD8^+^ T cells, NK cells, Tregs, myeloid-interacting immune states	Reinforce inhibitory tone through ligand systems including CEACAM1, phosphatidylserine, CD155, and CD112; modulate CD226-dependent activation	Co-expression with PD-1 may distinguish deeply engaged tumor-reactive immunity from simple receptor abundance, but excessive terminal exhaustion may limit durable rescue	Broad TIM-3/TIGIT-positive immune activation may mark patients at risk of exaggerated immune remodeling under multi-checkpoint blockade	Huang et al. (14), *Nature*, 2015; Johnston et al. (42), *Cancer Cell*, 2014; Fourcade et al. (43), *Journal of Experimental Medicine*, 2010.
VISTA and myeloid inhibitory programs	Tumor-associated macrophages, myeloid-derived suppressor cells, naïve or regulatory T-cell compartments	Maintains restraint in myeloid-rich environments and limits excessive antigen-presenting-cell activation	VISTA-rich myeloid tumors may be resistant to T-cell-directed PD-1 blockade alone and may require myeloid-contextualized strategies	Myeloid checkpoint disruption may amplify inflammatory tissue injury, particularly in tumors or hosts with high baseline innate activation	Wang et al. (43), *Journal of Experimental Medicine*, 2011;
NKG2A–HLA-E axis	NK cells and selected CD8^+^ T-cell subsets	Restrains cytotoxicity toward HLA-E-expressing cells and preserves NK-cell tolerance	NKG2A^+^ NK/CD8^+^ states may identify tumors exploiting HLA-E-mediated immune escape and may support NKG2A-directed combinations	Releasing NK-cell and CD8^+^ cytotoxicity may increase risk when normal tissues express protective HLA-E-dependent tolerance signals	Salomé et al. (24), *Cancer Cell*, 2022.
Activating receptor and cytokine counterweight	CD3/TCR signaling, CD28 co-stimulation, CD226 axis, IL-2, IL-7, IL-15, type I IFNs, IFN-γ	Provides the activation “drive” that inhibitory receptors restrain; determines whether checkpoint release produces productive immunity or tissue injury	Checkpoint expression is most informative when paired with evidence of preserved co-stimulatory competence, proliferative reserve, and clonal tumor engagement	Toxicity becomes clinically relevant when inhibitory release occurs in the presence of strong cytokine tone, tissue inflammation, or autoreactive immune reserve	Tooley et al. (30), *Cell Reports Medicine*, 2024.

This table is intended to prevent overinterpretation of inhibitory receptor abundance alone. PD-1, programmed cell death protein 1; CTLA-4, cytotoxic T-lymphocyte-associated protein 4; LAG-3, lymphocyte activation gene 3; TIM-3, T-cell immunoglobulin and mucin-domain containing 3; TIGIT, T-cell immunoreceptor with Ig and ITIM domains; VISTA, V-domain Ig suppressor of T-cell activation; NKG2A, natural killer group 2A; TCR, T-cell receptor; MHC-II, major histocompatibility complex class II; FGL1, fibrinogen-like protein 1; CEACAM1, carcinoembryonic antigen-related cell adhesion molecule 1; NK, natural killer; Treg, regulatory T cell; IFN, interferon; irAE, immune-related adverse event.

### 2.4 From tumor reinvigoration to peripheral organ injury.

A mechanistic account that links antitumor efficacy to immune-related toxicity must explain how an intervention acting on immune cells produces injury in non-lymphoid organs. Although the overarching process is described as loss of tolerance, this shorthand conceals layered biology. Checkpoint blockade lowers activation thresholds, drives clonal expansion, restores effector programs, redirects trafficking, and weakens regulatory control (17, 19, 20). These same alterations prove beneficial when they reactivate dormant or restrained tumor-reactive lymphocytes within neoplastic lesions (12). They prove harmful when identical programs encounter shared self-antigens, pre-existing inflammatory niches, or latent autoreactive repertoires in normal tissues (16, 38).

Distinct organs illustrate tissue-specific variants of this principle. In checkpoint-induced colitis, activated tissue-resident and effector T cells accumulate in the mucosa, yet the dominant drivers differ by regimen: CTLA-4 blockade more prominently disrupts regulatory T-cell function and elicits broad mucosal activation, whereas PD-1 blockade more characteristically unleashes tissue-resident cytotoxic populations (16, 38, 44). Clinical and mechanistic studies indicate that endocrine toxicities, including thyroiditis, autoimmune diabetes and hypophysitis, arise from the interplay between pre-existing organ-specific autoantibodies and the differential effects of PD-1 versus CTLA-4 blockade on peripheral tolerance and regulatory T-cell compartments, resulting in distinct incidence patterns across regimens (39, 40, 45). In myocarditis and pneumonitis, PD-1 appears particularly important for restraining tissue-resident populations that, once reactivated, can rapidly mediate destructive inflammation (46–48). These adverse events are therefore not random complications but predictable, tissue-specific failures of homeostatic regulation.

This perspective also clarifies why efficacy and toxicity, although correlated, are not identical. Both originate from successful pharmacologic engagement of previously restrained immune circuits (5, 29). The variables that steer those circuits toward tumor eradication versus organ damage, however, overlap only partially. Tumor-intrinsic factors—antigenicity, clonal fitness, antigen presentation efficiency, and local immune architecture—predominantly govern efficacy (5, 49). Host factors—HLA background, pre-existing autoantibodies, tissue-resident memory pools, regulatory reserve, and intrinsic organ susceptibility—predominantly shape toxicity. Inhibitory receptor profiling acquires clinical value precisely because it intersects both domains; its interpretive power, however, emerges only when these additional biological layers are incorporated into the analytical framework (5, 29, 49).

## 3 Profiling inhibitory receptor states in tumors and blood

### 3.1 Tissue-based pathology and spatially resolved assays

Tumor tissue remains the most immediate substrate for dissecting the local logic of checkpoint dependence. Conventional immunohistochemistry already informs clinical decisions through PD-L1 testing, and multiplex immunohistochemistry or immunofluorescence extends this capability by enabling simultaneous detection of checkpoint molecules, lineage markers, and stromal architecture in formalin-fixed material (28, 32, 50). The distinctive advantage of these methods lies in the retention of spatial context. Whether PD-1-positive cells lie adjacent to tumor nests, reside within stroma, cluster in tertiary lymphoid structures, or localize to suppressive myeloid zones shapes the biological interpretation of the biomarker at least as much as staining intensity (51, 52).

Spatial information proves especially valuable because identical overall densities can mask fundamentally different states. A lesion densely populated by PD-1-positive T cells may be inflamed and therapeutically responsive if those cells are tumor-reactive and appropriately positioned relative to malignant epithelium (13, 28, 32). The same “checkpoint-rich” profile may instead reflect immune exclusion, nonspecific bystander recruitment, or suppressive cellular clustering. Spatial pathology therefore refines both mechanistic insight and clinical utility (28, 32). It can separate inflamed microenvironments from myeloid-dominant or immune-excluded tumors, identify tertiary lymphoid structures associated with favorable outcomes, and flag checkpoint-positive regulatory populations that may predict benefit from combination regimens while also signaling heightened toxicity risk (28, 52).

The limitations of tissue-based assays are equally apparent. Sampling bias is inherent, particularly with small biopsies. Receptor expression varies across lesions and can shift rapidly once treatment begins. Scoring remains sensitive to antibody choice, staining protocols, platform-specific cut-offs, and observer variability, although ongoing standardization efforts have narrowed these sources of discordance (10, 11, 53). Most critically, tissue assays capture only static snapshots. In a system as pharmacodynamically fluid as checkpoint blockade, their principal contribution may lie in serving as the pretreatment reference point within a larger longitudinal strategy rather than functioning as independent diagnostic tools.

### 3.2 Peripheral blood profiling and longitudinal immune monitoring

Peripheral blood offers complementary strengths. Although it lacks the spatial and tumor-specific resolution of tissue, it is minimally invasive, readily repeatable, and ideally suited to serial monitoring. Multicolor flow cytometry and mass cytometry can enumerate checkpoint-bearing populations across CD8 T cells, CD4 subsets, regulatory T cells, natural killer cells, and myeloid lineages. Because these platforms resolve co-expression patterns and activation markers at single-cell level, they are well positioned to define composite immune states rather than isolated receptor abundances.

Blood-based profiling has produced some of the most reproducible pharmacodynamic correlates of immunotherapy activity. Early expansion of PD-1-positive CD8 T cells following anti-PD-1 treatment, frequently quantified by Ki-67 expression, has been consistently linked to subsequent clinical benefit (14, 26, 35). Higher-dimensional analyses further indicate that on-treatment immune remodeling often carries greater predictive weight than baseline measurements, particularly when the readout tracks the transition from restrained to functionally productive effector states rather than static receptor density (54, 55). These findings support an adaptive monitoring approach: absence of early reinvigoration substantially lowers the probability of later durable response.

Blood monitoring is likewise well suited to early toxicity detection. The same technologies can identify expansion of activated checkpoint-positive populations, shifts in regulatory cell frequencies, or movement toward inflammatory immunotypes before clinical organ injury becomes evident (56, 57). Nevertheless, blood carries intrinsic constraints. Certain immune populations that drive decisive outcomes reside primarily in tissues and are underrepresented in circulation (54, 58). A blood signature of activation may indicate tumor control, evolving toxicity risk, or both. Interpretation therefore gains greatest reliability when blood readouts are explicitly anchored to the specific regimen, sampling time point, and pretreatment tissue context.

### 3.3 Single-cell and spatial multi-omics

Single-cell transcriptomics, T-cell receptor sequencing, and spatially resolved multi-omics have substantially refined mechanistic understanding of checkpoint biology. These approaches demonstrate that exhaustion constitutes a progressive trajectory rather than a fixed endpoint; that tumor-specific and bystander T cells can be separated by clonality and transcriptional programs; and that checkpoint-regulated states arise within discrete microanatomical niches rather than uniformly throughout a lesion (13, 15, 59). They further uncover receptor combinations not readily apparent from protein staining and delineate lineage relationships among precursor, transitional, terminal, regulatory, and innate populations.

For translational biomarker development, the primary near-term value of these platforms lies in signature derivation rather than immediate clinical deployment. They highlight which receptor modules, transcriptional programs, and clonal architectures merit reduction to practical assay formats. The distinction between TCF1-associated precursor states and terminal TOX-high, multi-checkpoint states, for example, originated in deep mechanistic studies and now guides interpretation of routine flow cytometric and tissue-panel data (60, 61). Spatial analyses have similarly clarified why tertiary lymphoid structures and immune hubs can remain favorable even when inhibitory receptor expression is elevated (28, 32).

At present these technologies remain too expensive, technically variable, and computationally intensive for widespread routine application. They demand fresh tissue, standardized preprocessing, rigorous batch correction, and specialized analytical pipelines. Their most immediate clinical contribution is therefore indirect: generation of distilled biomarker panels, definition of mechanistic subtypes, and creation of reference maps against which simpler pathology- and blood-based assays can be calibrated and interpreted.

### 3.4 Clinically accessible translational surrogates

Although single-cell and spatial multi-omics platforms have yielded critical mechanistic insights into inhibitory receptor states and exhaustion trajectories, these discovery technologies remain resource-intensive, technically variable, and computationally demanding, thereby limiting immediate widespread clinical deployment; this has driven focused efforts to distill key transcriptional programs, receptor modules, and clonal architectures into simpler, clinically accessible assay formats (61–63). This concern is well founded. If inhibitory receptor profiling is to influence everyday decision-making, it must ultimately map onto measurements obtainable in routine hospital laboratories (63, 64). Several practical bridges already exist. Standard pathology services can accommodate limited multiplex tissue panels centered on PD-1, LAG-3, TIGIT, CTLA-4, CD8, FOXP3, and myeloid markers (63, 65). Standardized blood cytometry platforms can quantify selected checkpoint-positive subsets over time without requiring full discovery-scale immunophenotyping (64). Organ-specific laboratory tests, autoantibody panels, complete blood counts, inflammatory indices, and plasma protein signatures supply additional layers of information on host susceptibility or early pharmacodynamic shifts (**Table 2**).

**Table 2:** Translational platforms and clinically accessible surrogates for profiling inhibitory receptor states.

Platform or modality	Typical sample and timing	Most informative readouts	Principal strengths	Main limitations	Likely translational role	Reference
Immunohistochemistry and multiplex immunofluorescence	Pretreatment or on-treatment FFPE tumor tissue	PD-1, PD-L1, CTLA-4, LAG-3, TIGIT, TIM-3, VISTA, CD8, FOXP3, myeloid markers; spatial localization in tumor nests, stroma, invasive margin, and TLS	Compatible with routine pathology; preserves tissue architecture; supports spatial interpretation of receptor-positive immune states	Semi-quantitative; sensitive to antibody choice, scoring method, and tissue sampling; limited longitudinal feasibility	Most practical bridge from mechanistic receptor biology to clinically deployable tissue biomarkers	Taube et al. (33), *Journal for ImmunoTherapy of Cancer*, 2025; Anagnostou et al. (53), *Cancer Epidemiology, Biomarkers & Prevention*, 2010.
Flow cytometry and mass cytometry	Peripheral blood at baseline and early on-treatment time points; fresh tumor digests when available	PD-1^+^Ki-67^+^ CD8^+^ T cells, PD-1^+^LAG-3^+^ or PD-1^+^TIM-3^+^ T cells, TIGIT^+^ NK cells, checkpoint-positive Tregs, activation-to-exhaustion transitions	Single-cell protein-level quantification; suitable for serial monitoring; captures dynamic pharmacodynamic remodeling	Requires viable cells, standardized processing, harmonized panels, and robust computational analysis	Core modality for longitudinal immunomonitoring and dynamic benefit–risk modeling	Finak et al. (66), *Scientific Reports*, 2016; Gadalla et al. (67), *Frontiers in Oncology*, 2019.
Single-cell RNA sequencing and TCR-linked multi-omics	Fresh tumor tissue or PBMCs, usually in discovery or translational cohorts	Precursor versus terminal exhaustion, tumor-reactive clonotypes, receptor co-expression modules, lineage transitions, fixed dysfunction signatures	Resolves cellular states and clonal specificity beyond marker abundance; useful for defining mechanistic biomarker modules	Expensive; technically demanding; not yet suited for routine hospital deployment; vulnerable to batch and preprocessing effects	Discovery platform for deriving simplified receptor-state panels and reference maps	Komuro et al. (34), *Journal for ImmunoTherapy of Cancer*, 2023; Tooley et al. (30), *Cell Reports Medicine*, 2024; Kim et al. (68), *Journal for ImmunoTherapy of Cancer*, 2025.
Spatial transcriptomics and spatial proteomics	Archival or fresh tumor sections, baseline or paired translational samples	Microanatomical location of checkpoint-expressing effector, regulatory, NK, and myeloid populations; proximity to tumor cells, TLS, vessels, and exclusion barriers	Couples molecular depth with tissue architecture; distinguishes inflamed, excluded, myeloid-rich, and TLS-associated receptor states	High cost, limited throughput, variable spatial resolution, and heavy analytical requirements	Next-generation tissue classifier; currently strongest for mechanistic refinement and panel design	Xu et al. (69), *Cancer Immunology, Immunotherapy*, 2025; Anagnostou et al. (70), *Cell Reports Medicine*, 2020
Routine laboratory, plasma, and organ-surveillance markers	Baseline and serial blood testing during treatment	CBC-derived indices, NLR, ALC, inflammatory chemokines, plasma protein signatures, autoantibodies, thyroid function, troponin, natriuretic peptides, liver enzymes	Clinically accessible; inexpensive; repeatable; directly relevant to toxicity surveillance	Limited specificity; often reflects systemic inflammation rather than receptor biology alone	Required clinical layer for making receptor-based models usable outside specialized centers	Jing et al. (41), *Nature Communications*, 2020; Johannet et al. (39), *Clinical Cancer Research*, 2022; Ayoub et al. (71), *JACC: Advances*, 2025.
Imaging, digital pathology, and host-immune reserve	Routine CT, MRI or PET imaging; digital H&E pathology; age- and immune-reserve measures	Tumor burden, lesion distribution, early radiographic kinetics, machine-readable immune architecture, thymic activity, naïve T-cell reserve, recent thymic emigrant surrogates	Clinically scalable; captures tumor and host context; may integrate naturally with electronic health records	Indirect readouts; requires prospective validation and harmonized computational pipelines	Practical contextual layer for regimen selection, risk adjustment, and host-centered prediction	Rakaee et al. (72), *JAMA Oncology*, 2025; Kao et al. (73), *Nature Communications*, 2025; Bernatz et al. (74), *Nature*, 2026.

This table separates discovery platforms from clinically deployable surrogates. FFPE, formalin-fixed paraffin-embedded; PD-L1, programmed death ligand 1; FOXP3, forkhead box P3; TLS, tertiary lymphoid structure; TCR, T-cell receptor; PBMC, peripheral blood mononuclear cell; CBC, complete blood count; NLR, neutrophil-to-lymphocyte ratio; ALC, absolute lymphocyte count; CT, computed tomography; MRI, magnetic resonance imaging; PET, positron emission tomography; H&E, hematoxylin and eosin.

Clinical and imaging surrogates are equally relevant. Baseline tumor burden, lesion distribution, and early radiographic kinetics remain imperfect yet practical descriptors of disease context. Digital pathology combined with machine-learning algorithms can extract immune architectural features from routine histologic slides, while multimodal prediction models that integrate standard clinical variables, electrocardiography, imaging, and laboratory biomarkers have begun to demonstrate utility for specific toxicities such as myocarditis (71, 72, 75). These accessible measures do not supplant receptor biology; they provide the necessary clinical frame within which receptor data acquire meaning. A checkpoint-rich signature interpreted without reference to tumor burden, patient age, comorbidities, laboratory trajectory, and treatment regimen is less informative than a modest receptor panel embedded in a clinically grounded model (71, 72, 75).

An emerging and biologically compelling notion in this context is that of host immune reserve. Preserved naïve T-cell pools, age-associated immune remodeling, and the broader concept of thymic competence may influence both the ability to recruit new clonotypes and the probability that checkpoint release will expand autoreactive populations (49, 73, 74). Although direct clinical assays of thymic function are not yet standardized within immuno-oncology, the underlying idea merits prospective investigation. In current practice it can be approximated indirectly through patient age, naïve T-cell composition, markers of recent thymic emigrants, and other host-level immune parameters (73, 74).

## 4 Predicting antitumor efficacy from inhibitory receptor context

### 4.1 The value and limits of single-receptor biomarkers

The most straightforward biomarker model posits that elevated expression of a particular inhibitory receptor forecasts benefit from its pharmacologic blockade. PD-L1 testing exemplifies this logic and retains utility in selected clinical settings. Its shortcomings, however, are well documented. Objective responses occur in tumors with low or absent PD-L1 expression, and many PD-L1-high tumors prove refractory (4, 76, 77). Parallel caveats apply to PD-1 expression on tumor-infiltrating lymphocytes. Elevated PD-1 can identify recently activated, tumor-reactive cells, yet it can equally mark bystander populations or cells whose dysfunction has advanced beyond rescue (32, 34). Consequently, single-receptor measurements generally enrich for response without reliably determining outcome.

These markers nevertheless retain utility when placed in appropriate context. Substantial tissue expression of PD-1 or LAG-3 can indicate that the corresponding pathway is actively engaged, whereas negligible expression may imply limited pharmacologic relevance. In practice this distinction helps differentiate truly immune-cold lesions from those in which restraint exists but is inadequately captured by PD-L1 assessment alone (77, 78). Similarly, a post-treatment rise in proliferating PD-1-positive CD8 T cells in blood is not interchangeable with baseline PD-1 abundance; it registers a drug-induced transition in cellular state and therefore lies closer to the mechanistic basis of efficacy (14, 26, 35). Static and dynamic readouts must be distinguished.

Single-receptor biomarkers are therefore most productively regarded as entry points into more comprehensive models. They are informative when the question concerns whether a given pathway is plausibly operative, but less decisive when the question concerns whether a patient will achieve durable tumor control under a specific regimen. For the latter purpose, the combinatorial architecture of inhibitory receptor states carries greater weight than any individual receptor in isolation (13, 15, 78).

### 4.2 Co-expression modules and the exhaustion continuum

High-dimensional analyses have established that checkpoint biology is modular. The clinically salient signal frequently resides not in PD-1, TIGIT, or LAG-3 considered separately, but in the cellular state defined by their coordinated expression together with transcriptional regulators, proliferative capacity, and clonal specificity (31, 78). This principle is clearest within the exhausted CD8 T-cell compartment. Precursor-like cells typically express PD-1 with limited additional inhibitory receptors, maintain TCF1-associated self-renewal potential, and generate the proliferative burst observed after PD-1 blockade (13, 15, 78). Transitional and terminal states progressively acquire TIM-3, LAG-3, TIGIT, TOX-linked transcriptional programs, and progressive metabolic and epigenetic fixation.

**Figure 3:**
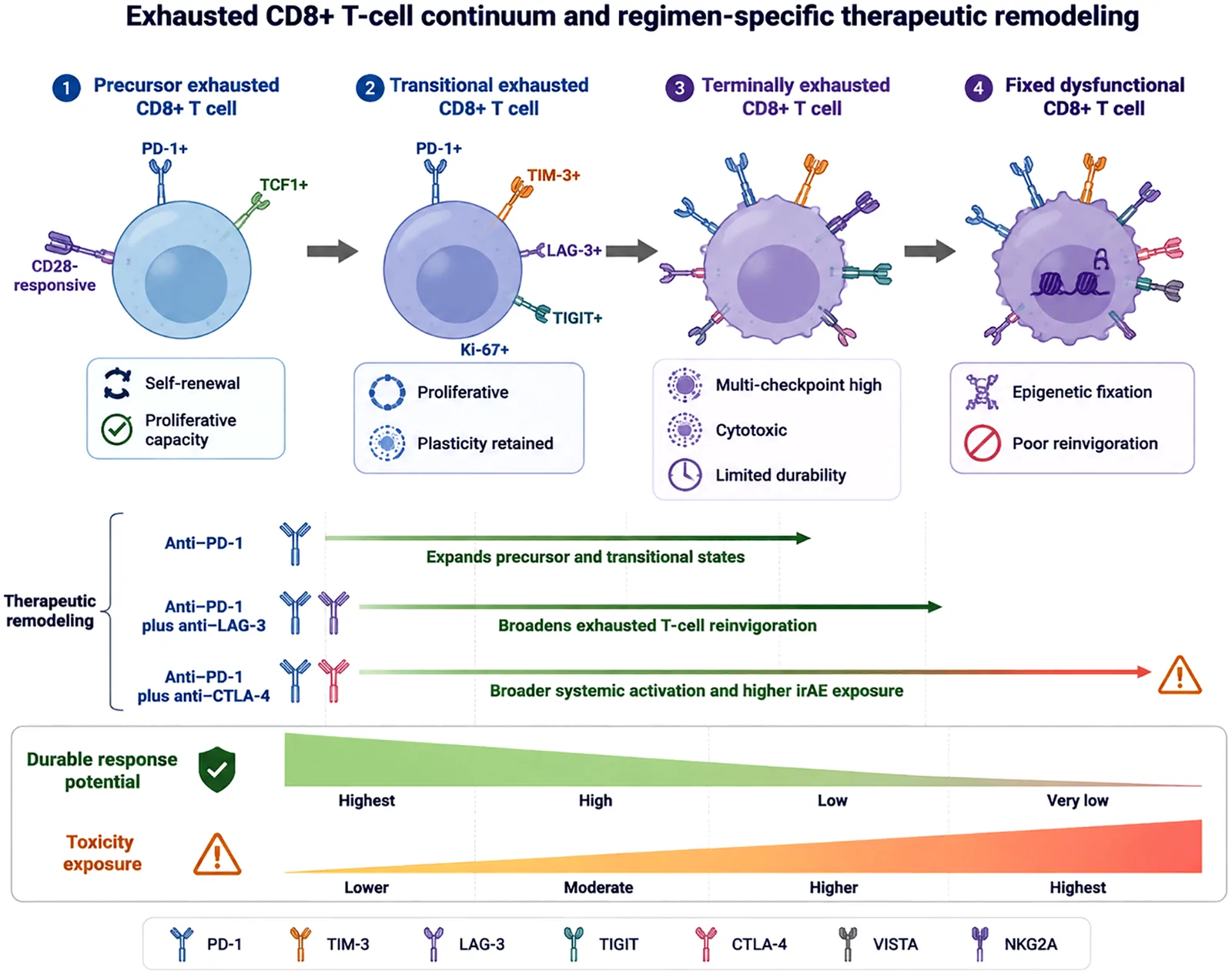
Exhausted CD8+ T-cell continuum and regimen-specific therapeutic remodeling. The diagram traces CD8+ T-cell dysfunction from PD-1+TCF1+ precursor-exhausted cells with CD28 responsiveness and proliferative capacity, through proliferative transitional states, to multi-checkpoint-high terminal exhaustion and epigenetically fixed dysfunction. Anti-PD-1 preferentially expands precursor and transitional populations; combined PD-1/LAG-3 blockade may broaden exhausted T-cell reinvigoration; and PD-1/CTLA-4 blockade produces wider systemic activation with greater toxicity exposure. Durable response potential falls as fixation increases, whereas toxicity exposure rises with broader immune release.

This continuum accounts for otherwise puzzling clinical observations. Tumors enriched for checkpoint-positive effector populations may respond favorably because they harbor pre-existing tumor-reactive immunity. Tumors dominated by terminally exhausted states, however, may fail to sustain benefit because reinvigoration remains transient and is not replenished from progenitor pools (12, 13, 78). Composite modules therefore outperform binary assessments of checkpoint positivity. The burden of stem-like precursors, the architecture of co-expression, T-cell receptor clonality, and the ratio of reinvigoration to tumor burden together provide a more accurate reflection of checkpoint sensitivity than PD-L1 or PD-1 measurement alone (**Figure 3**).

Spatial organization further refines this picture. Checkpoint-high T cells situated within tertiary lymphoid structures or immediately adjacent to tumor nests can indicate productive, organized immunity, whereas comparable cells sequestered behind exclusion barriers may reflect ineffective engagement (28, 32, 33). The most informative efficacy biomarkers therefore integrate state with location: which checkpoint-bearing cells are present, what differentiation state they occupy, and where they reside within the tumor ecosystem.

### 4.3 Regimen-specific interpretation

No inhibitory receptor profile carries an invariant meaning across treatment regimens. A pattern that predicts sensitivity to PD-1 monotherapy may indicate insufficient pathway release for meaningful benefit in a tumor that would respond better to dual checkpoint blockade (44, 79, 80). The identical pattern may instead forecast excess toxicity if applied to an intensive combination regimen.

These distinctions arise from the divergent mechanisms of the available agents. Anti-PD-1 therapy primarily expands and reinvigorates exhausted or precursor-like effector populations already resident within tumors. Anti-CTLA-4 exerts stronger effects at the priming stage and on regulatory compartments, producing broader systemic immune activation (44, 79, 80). PD-1 plus LAG-3 blockade appears particularly relevant in tumors enriched for co-expressing exhausted T cells, whereas PD-1 plus CTLA-4 blockade may be preferred when broader immune activation or regulatory perturbation is required. These are not interchangeable therapeutics, and biomarkers cannot remain regimen-agnostic if the drugs themselves are not (78, 79).

The practical consequence is clear: inhibitory receptor measurements must be interpreted relative to the intended regimen. A profile that supports target availability under PD-1 monotherapy may not justify addition of CTLA-4 blockade when toxicity liability is elevated. Conversely, a tumor displaying advanced co-inhibitory architecture and limited expected response to PD-1 alone may still represent a rational candidate for combination therapy provided host risk appears acceptable. Biomarker development should therefore advance in tandem with regimen class rather than pursue a universal checkpoint score (**Table 3**) (78–80).

**Table 3:** Practical interpretation of inhibitory receptor profiles across clinical settings and treatment regimens.

Clinical setting	Regimen context	Informative receptor-state features	Possible efficacy signal	Possible toxicity signal	Decision-making implication	Reference
Metastatic melanoma or other highly ICI-responsive tumors	Anti-PD-1 monotherapy versus PD-1/CTLA-4 or PD-1/LAG-3 combinations	PD-1^high exhausted CD8^+^ T cells, LAG-3 or TIM-3 co-expression, checkpoint-positive Tregs, early PD-1^+^Ki-67^+^ CD8^+^ expansion	Reinvigoratable exhausted immunity may support high response probability, especially when tissue architecture is inflamed or TLS-associated	Broad co-activation or regulatory destabilization may increase multisystem irAE risk under combination therapy	Select monotherapy when benefit is adequate; reserve combination therapy for receptor-state patterns suggesting insufficient PD-1-only release	Tawbi et al. (79), *New England Journal of Medicine*, 2022; Miller et al. (13), *Nature Immunology*, 2019; Gide et al. (80), *Cancer Cell*, 2019.
Non-small cell lung cancer with variable inflammatory status	Anti-PD-1/PD-L1 alone, chemo-immunotherapy, or selected dual-checkpoint strategies	PD-L1 context plus PD-1^+^ or TIGIT^+^ CD8^+^ T-cell states in tumor and blood; early proliferative CD8^+^ response	Inflamed tumors with tumor-reactive exhaustion and early blood reinvigoration are more likely to benefit from PD-1-based therapy	Intensified regimens may increase toxicity exposure, particularly with rapid systemic immune activation or poor organ reserve	Move beyond a one-size-fits-all PD-L1 threshold by integrating receptor state, dynamic monitoring, and clinical risk	Mazzaschi et al. (81), *Clinical Cancer Research*, 2018; Dutta et al. (35), *Cancer Immunology, Immunotherapy*, 2025; Kamphorst et al. (82), *PNAS*, 2017.
Curative-intent neoadjuvant or adjuvant therapy	Short-course preoperative ICI or postoperative maintenance	Baseline tissue receptor landscape, TLS maturity, precursor-like CD8^+^ states, early pharmacodynamic activation	A favorable receptor-state profile may identify patients likely to derive recurrence-risk reduction	Even moderate grade ≥3 irAE risk may be unacceptable when baseline curability is high	Apply stricter benefit thresholds before escalating to combination or high-intensity regimens	Xu et al. (69), *Cancer Immunology, Immunotherapy*, 2025; Johannet et al. (39), *Clinical Cancer Research*, 2022; Weber et al. (83), *Journal of Clinical Oncology*, 2023.
Patients with autoimmune vulnerability, major comorbidity, or low organ reserve	Any ICI regimen, especially CTLA-4-containing or multi-checkpoint blockade	Checkpoint expression on Tregs and activated effector subsets; systemic activation at baseline; naïve T-cell and thymic-reserve indicators	Benefit may persist, but interpretation must weigh target engagement against risk of treatment interruption or immunosuppression	Lower tolerance reserve may convert moderate immune activation into severe or organ-threatening irAEs	Prefer monotherapy, organ-specific surveillance, and early de-escalation when predicted harm exceeds incremental benefit	Marks et al. (49), *JCI Insight*, 2026; Kao et al. (73), *Nature Communications*, 2025; Bernatz et al. (74), *Nature*, 2026.
Early on-treatment monitoring during the first one to two cycles	Applicable across PD-1, PD-L1, CTLA-4, LAG-3, and combination regimens	Serial changes in receptor-positive T-cell and NK-cell subsets; emergence of activated checkpoint-positive clones; divergence between blood and tissue signals	Early expansion of reinvigorated effector populations may support continuation before radiographic benefit is visible	Abrupt activation plus laboratory or symptom change may indicate impending irAE before full clinical manifestation	Enable adaptive management: continue, intensify surveillance, de-escalate, or intervene before severe toxicity	Kamphorst et al. (82), *PNAS*, 2017; Nuñez et al. (84), *Med*, 2023.
Immune-cold or target-poor tumors	PD-1/PD-L1 monotherapy often insufficient; combination or non-ICI strategies may be considered	Low TIL density, low PD-1/PD-L1 target availability, myeloid-rich or VISTA-positive suppressive architecture, absent early blood reinvigoration	Low likelihood of durable benefit from checkpoint blockade alone	Low immune activation may imply lower irAE risk, but added combinations may introduce toxicity without adequate benefit	Avoid escalation based solely on checkpoint negativity; consider alternative priming, myeloid-targeted, or non-immunotherapy approaches	Wang et al. (23), *Journal of Experimental Medicine*, 2011; Anagnostou et al. (70), *Cell Reports Medicine*, 2020; Rakaee et al. (72), *JAMA Oncology*, 2025.

This table provides scenario-based interpretation rather than prescriptive treatment rules. ICI, immune checkpoint inhibitor; PD-1, programmed cell death protein 1; PD-L1, programmed death ligand 1; CTLA-4, cytotoxic T-lymphocyte-associated protein 4; LAG-3, lymphocyte activation gene 3; TIM-3, T-cell immunoglobulin and mucin-domain containing 3; TIGIT, T-cell immunoreceptor with Ig and ITIM domains; Treg, regulatory T cell; TLS, tertiary lymphoid structure; TIL, tumor-infiltrating lymphocyte; NK, natural killer; irAE, immune-related adverse event.

### 4.4 Host and tumor modifiers that sharpen prediction

Even well-characterized receptor states require additional contextual modifiers. Tumor-intrinsic variables include mutational burden, antigen presentation competence, IFN-γ-responsive transcriptional programs, maturity of tertiary lymphoid structures, myeloid composition, and the distinction between inflamed and excluded architectures (73, 74, 85). Host-intrinsic variables encompass age, systemic inflammatory tone, microbiome composition, prior immune injury, and baseline immune competence. These factors do not merely introduce noise; they actively shape the biological interpretation of checkpoint states themselves (74, 85).

Inhibitory receptor states must be interpreted within tumor-intrinsic and host-intrinsic context. The same co-inhibitory profile may indicate a restrained yet reinvigoratable antitumor response in one patient, yet reflect chronic inflammatory overdrive with limited additional therapeutic gain in another; in still others, scant checkpoint expression may signify insufficient T-cell recruitment or a dominant myeloid barrier rather than absence of immune relevance (74, 78, 85).

For efficacy prediction, the most promising strategy is accordingly layered rather than reductive. Baseline tissue context identifies targetable immune architecture; selective blood monitoring captures pharmacodynamic engagement; and host-level modifiers estimate whether the observed immune state can be translated into durable tumor control. Inhibitory receptor profiling occupies a central position within this framework, yet it functions most effectively when embedded among these complementary elements rather than standing alone (71, 72, 78).

## 5 Predicting immune-related toxicity and defining the benefit-risk continuum

### 5.1 Baseline susceptibility and immune homeostatic reserve

Pre-existing host susceptibility is frequently the most consequential yet least visible determinant of immune-related toxicity. Checkpoint inhibitors do not generate autoreactivity *de novo*; they expose, amplify, or redirect immune programs that were already latent, restrained, or clinically silent (5, 16, 29). The relevant biological substrate encompasses organ-specific autoantibodies, permissive HLA alleles, antecedent autoimmune disease, diminished regulatory T-cell reserve, tissue-resident memory populations, and inflammatory microbiome configurations. Toxicity therefore represents the outcome of applying a defined degree of checkpoint release to a host possessing a particular tolerance reserve rather than an incidental on-treatment event (86, 87).

Baseline inhibitory receptor states intersect with this reserve without fully defining it. Certain studies link severe immune-related adverse events to broader naïve or less constrained baseline immune repertoires, whereas others implicate pre-existing checkpoint-positive effector pools that become poised for rapid expansion once therapy begins (84, 86, 87). These observations are not inherently contradictory; they likely describe distinct mechanistic routes to toxicity. One route originates from insufficient homeostatic restraint coupled with substantial capacity for immune diversification. Another originates from abundant suppressed effectors that undergo abrupt activation when dominant checkpoints are removed. The direction of the association probably varies with regimen, underlying disease, sampling timing, and the specific immune compartment under study (86, 87).

Emerging evidence suggests that thymic health and overall immune reserve may influence both antitumor efficacy and toxicity risk during checkpoint blockade. Preserved thymopoiesis and expanded naïve T-cell compartments can support more adaptable antitumor responses, while potentially increasing the autoreactive repertoire available for off-target activation upon potent checkpoint release (49, 73). In contrast, immunological aging is associated with diminished plasticity, heightened myeloid inflammation, and greater organ vulnerability. These associations remain incompletely characterized and warrant prospective validation; nevertheless, they underscore the necessity of incorporating host homeostatic biology into toxicity prediction models from the outset, rather than treating immune-related adverse events solely as a secondary consequence of antitumor efficacy (49, 87).

### 5.2 Early on-treatment dynamics and organ-selective toxicity

For many patients the most informative toxicity biomarkers are dynamic rather than static. Immune-related adverse events commonly emerge after an interval of clinically silent immune remodeling during which activated T-cell populations expand, cytokine profiles shift, and tissue-specific damage accumulates before symptoms appear (41, 84). Longitudinal monitoring therefore holds particular promise for early prediction.

The most readily detectable signal is accelerated systemic activation. Rapid expansion of checkpoint-positive effector populations, surges in inflammatory chemokines, or abrupt shifts toward activated blood immunotypes can identify patients crossing from productive reinvigoration into hazardous immune overdrive (16, 84). Toxicity, however, is not solely a question of magnitude. Organ-selective biology is decisive. Checkpoint-induced colitis engages colonic immune circuits distinct from those driving thyroiditis, hypophysitis, myocarditis, or vitiligo (38, 46). Tissue-resident memory T cells figure prominently in certain toxicities, whereas autoantibodies or endocrine predisposition predominate in others.

Checkpoint receptor dynamics on circulating T cells may provide additional pharmacodynamic signals. Following PD-1 blockade, surface PD-1 expression frequently declines, consistent with receptor occupancy or internalization, while selected alternative inhibitory receptors (e.g., LAG-3 or TIGIT) show compensatory upregulation in a subset of patients. These patterns have been interpreted as reflecting either effective containment of immune activation or incomplete regulatory compensation; however, their specific utility for distinguishing self-limited remodeling from impending organ injury remains incompletely defined and merits dedicated prospective investigation (13, 17, 18). A delayed or inadequate upregulation of secondary checkpoints, for example, may help distinguish self-limited immune activation from uncontrolled tissue injury (41, 84).

### 5.3 Clinically deployable markers beyond receptor panels

A clinically useful toxicity framework cannot depend exclusively on specialized receptor assays. Several more readily deployable markers already carry supporting evidence. Routine blood counts and derived indices such as the neutrophil-to-lymphocyte ratio have been associated with endocrine toxicity, myocarditis, and broader immune-related adverse event risk in retrospective series (88–90). Organ-specific autoantibodies can predict endocrine events, particularly thyroid dysfunction and autoimmune diabetes-related phenomena (39, 40). HLA genotypes linked to spontaneous autoimmunity also appear relevant to checkpoint toxicity in melanoma and lung cancer. These variables are attractive because they are obtainable before treatment and amenable to standardization across centers (**Table 4**) (86, 92).

**Table 4:** Receptor-informed modules for predicting immune-related adverse events.

irAE prediction domain	Mechanistic basis	Receptor-informed warning signal	Clinically accessible co-predictors	Practical implication	Reference
Systemic susceptibility to clinically significant irAEs	Checkpoint blockade lowers activation thresholds and may release autoreactive or broadly inflammatory immune circuits	High pretreatment checkpoint-positive effector pools, rapid expansion of activated CD8^+^ T cells, or imbalance between effector activation and regulatory restraint	Age, autoimmune history, baseline organ function, NLR, ALC, inflammatory proteins, HLA background, autoantibodies	Identify patients requiring intensified monitoring, avoidance of aggressive combinations, or early supportive-care planning	Jing et al. (41), *Nature Communications*, 2020; Kim et al. (68), *Journal for ImmunoTherapy of Cancer*, 2025.
Gastrointestinal toxicity and colitis	Disruption of mucosal tolerance, CTLA-4-dependent Treg control, and PD-1-regulated tissue-resident effector activity	Expansion or activation of checkpoint-positive mucosal or circulating effector T cells; inadequate compensatory inhibitory signaling during early inflammation	Baseline bowel disease, stool symptoms, CRP, fecal calprotectin where available, endoscopic threshold, microbiome-related features in research settings	Lower threshold for symptom-triggered evaluation; caution with CTLA-4-containing combinations in high-risk patients	Sasson et al. (38), *Gastroenterology*, 2021; Hailemichael et al. (29), *Cancer Cell*, 2022.
Endocrine toxicity	Loss of peripheral tolerance in thyroid, pituitary, or pancreatic islet tissues; contribution of pre-existing organ-specific autoimmunity	PD-1/CTLA-4 release in patients with autoreactive immune reserve; receptor readouts are most informative when paired with autoantibody status	Thyroid antibodies, endocrine autoantibodies, TSH, free T4, glucose, HbA1c, pituitary-axis testing when clinically indicated	Baseline endocrine screening and early laboratory surveillance, especially when autoantibodies or symptoms are present	Labadzhyan et al. (40), *Journal of Clinical Endocrinology & Metabolism*, 2022; Johannet et al. (39), *Clinical Cancer Research*, 2022.
Cardiac and pulmonary toxicity	Reactivation of tissue-resident memory T cells and systemic inflammatory amplification in organs with limited tolerance reserve	Rapid systemic T-cell activation, checkpoint-positive cytotoxic expansion, or inflammatory profiles discordant with tumor burden	Troponin, natriuretic peptides, ECG, echocardiography, chest CT, oxygen saturation, NLR/ALC shifts	Early cardiopulmonary risk stratification; low threshold for urgent evaluation when symptoms or biomarkers emerge	Drobni et al. (90), *Journal of the American Heart Association*, 2020; Ayoub et al. (71), *JACC: Advances*, 2025.
Cutaneous and neurologic toxicity	Shared antigen recognition, bystander inflammation, and breakdown of tissue-specific immune privilege	Activated checkpoint-positive effector populations, particularly when accompanied by systemic cytokine or autoantibody signals	Dermatologic examination, neurologic symptoms, creatine kinase where relevant, autoantibody testing in selected neurologic syndromes	Distinguish mild toxicity associated with effective immune activation from severe tissue injury requiring interruption or immunosuppression	Otsuka et al. (91), *Journal of Dermatology*, 2020.
Early on-treatment dynamic risk	Toxicity often emerges after pharmacodynamic immune remodeling rather than from baseline status alone	Abrupt expansion of PD-1^+^Ki-67^+^ CD8^+^ T cells, PD-1^+^LAG-3^+^ or PD-1^+^TIGIT^+^ subsets, or activated checkpoint-positive clones without adequate regulatory counterbalance	Serial CBC, chemokines, liver enzymes, endocrine tests, symptom-reported outcomes, early imaging or organ-specific tests when indicated	Convert static risk stratification into adaptive surveillance and timely intervention before severe organ injury develops	Nuñez et al. (84), *Med*, 2023; Kim et al. (68), *Journal for ImmunoTherapy of Cancer*, 2025.

This table is focused on irAE prediction rather than irAE management. irAE, immune-related adverse event; Treg, regulatory T cell; NLR, neutrophil-to-lymphocyte ratio; ALC, absolute lymphocyte count; HLA, human leukocyte antigen; CRP, C-reactive protein; TSH, thyroid-stimulating hormone; T4, thyroxine; HbA1c, glycated hemoglobin; ECG, electrocardiography; CT, computed tomography; CBC, complete blood count.

Plasma-based and proteomic signatures offer further refinement. Soluble inflammatory mediators, metabolic patterns, and multidimensional plasma signatures have shown promise for identifying older patients at elevated risk and for distinguishing biological pathways to toxicity that routine clinical variables cannot resolve (41, 71). High-dimensional blood immunoprofiling has likewise identified recurring immunotypes associated with differential outcomes, suggesting that simplified derivatives of these signatures may become clinically feasible (84, 93). Although none of these tools is yet ready for universal application, collectively they advance toxicity prediction beyond anecdotal risk factors.

Organ-specific surveillance also merits a more prominent position within integrated models (94, 95). Troponin, natriuretic peptides, electrocardiography, and targeted imaging are already employed in the evaluation of suspected myocarditis; thyroid function testing can detect evolving endocrinopathy before symptoms predominate; stool inflammatory markers and endoscopic criteria inform the management of gastrointestinal toxicity (40, 94). These assessments are frequently viewed as reactive diagnostics. They can, however, function predictively when embedded in serial monitoring frameworks that integrate baseline susceptibility with early biological deviation.

Finally, the clinical interpretation of toxicity biomarkers must account for therapeutic context. A given predicted probability of grade ≥3 immune-related adverse events carries different implications depending on disease setting: in metastatic disease, where durable tumor control may justify higher risk tolerance, versus adjuvant or curative-intent settings, where even moderate toxicity may be unacceptable given high baseline curability. Risk estimates that disregard this therapeutic context are therefore clinically incomplete and may lead to misaligned decision-making (5, 79, 83).

### 5.4 Integrated models for patient-specific therapeutic windows

**Figure 4:**
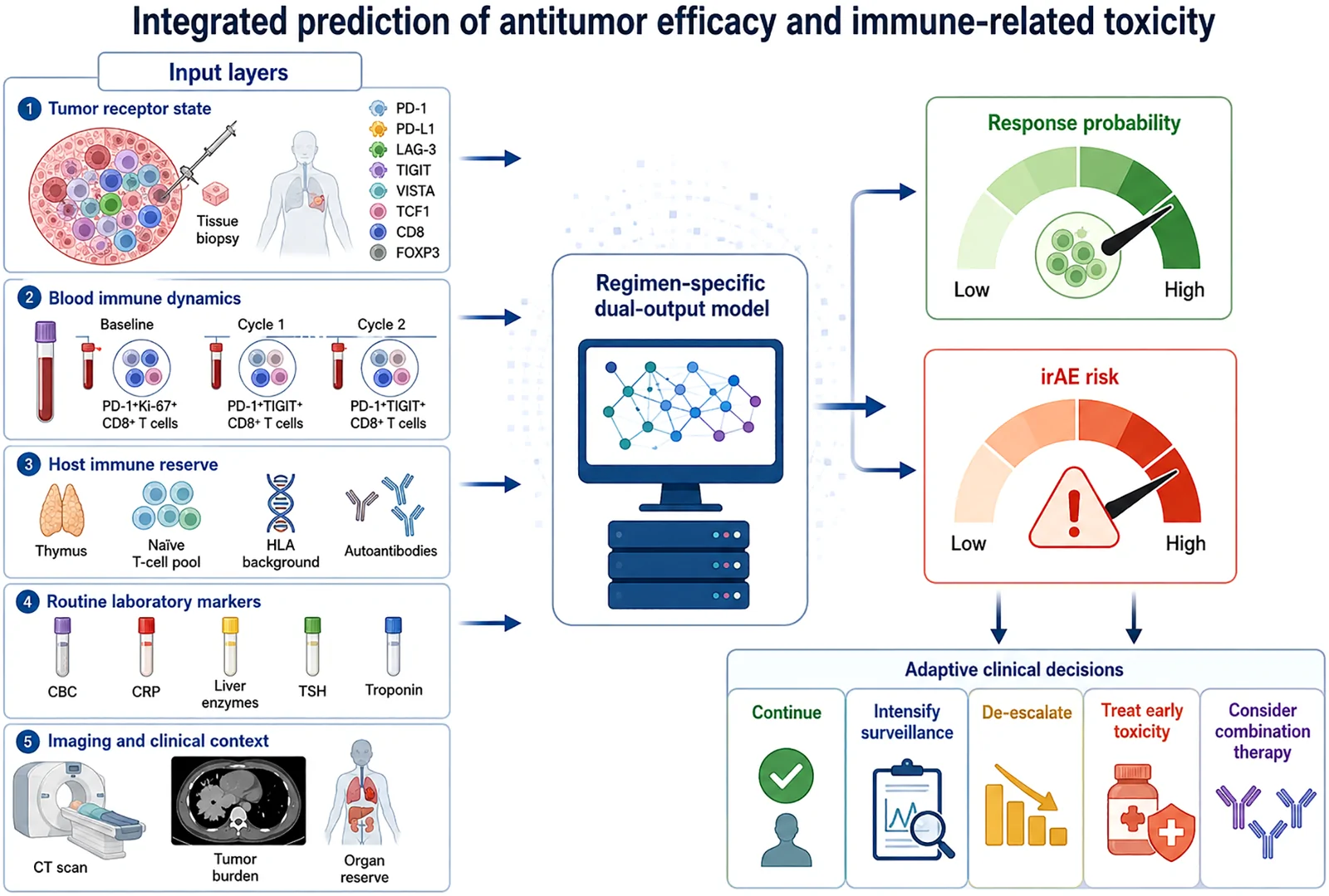
Integrated prediction of antitumor efficacy and immune-related toxicity. A regimen-specific dual-output model combines tumor receptor-state mapping, early blood immune dynamics, host immune reserve, routine laboratory markers, and imaging or clinical context. The model estimates response probability and immune-related adverse-event risk in parallel, translating layered biomarker information into adaptive clinical actions, including treatment continuation, intensified surveillance, de-escalation, early toxicity treatment or consideration of combination therapy.

The central clinical objective is not to optimize response biomarkers and toxicity biomarkers in isolation. It is to define a patient-specific therapeutic window in which checkpoint inhibition is sufficiently intense to produce durable tumor control yet sufficiently restrained to prevent unacceptable autoimmune injury (71, 72, 74). This window varies across tumors, regimens, and individual hosts, and inhibitory receptor profiling remains one of the few biomarker domains with plausible relevance to both sides of the equation (**Figure 4**).

The development of integrated, multi-task predictive frameworks that simultaneously estimate the probabilities of durable antitumor response and immune-related toxicity is therefore warranted. Informative inputs for such models encompass pretreatment tissue architecture, receptor co-expression modules, the balance between precursor and terminal exhaustion states, blood-based pharmacodynamic changes, inflammatory or proteomic markers, autoantibody status, HLA background, age, naïve T-cell reserve, microbiome features, and standard clinical variables (71, 72, 96). The output must be actionable. Examples include guidance toward monotherapy rather than combination treatment, intensification of organ-specific surveillance, escalation of early supportive care, or prompt transition to alternative therapy when both expected benefit and therapeutic target availability are low.

Machine-learning and multimodal approaches are increasingly appropriate for this task because the relevant interaction terms are biologically grounded. The principal challenge lies not only in predictive accuracy but in interpretability. Clinicians require clarity on whether a given risk estimate is driven primarily by a receptor-state signature, a host susceptibility marker, a laboratory trajectory, or an organ-specific warning pattern. Translational models that preserve mechanistic structure while delivering clinically transparent outputs may therefore prove more valuable than black-box algorithms trained on heterogeneous datasets (72, 96, 97).

Several principles should guide further development. The model must remain regimen-aware. Baseline and on-treatment information should be combined rather than treated as competing paradigms. Clinically accessible markers must be incorporated so that sophisticated biological insight is not confined to tertiary centers. Prospective validation is essential, as many current associations derive from modest retrospective cohorts. Intervention studies will ultimately be required; a biomarker that predicts toxicity acquires clinical meaning only when the prediction alters management in ways that preserve efficacy or reduce harm. Early evidence that targeted anti-inflammatory interventions, such as IL-6 blockade in selected contexts, may mitigate toxicity without abolishing antitumor activity renders this objective more attainable than previously appeared possible (29, 98, 99).

The broader implication is that inhibitory receptor profiling should not be positioned as a search for a single decisive test. Its genuine clinical value resides in helping to locate patients along a benefit-risk continuum and in continuously updating that location as treatment reshapes immune state. In this capacity it functions less as a static biomarker and more as a dynamic monitoring framework for precision immunotherapy.

## 6 Conclusion

Inhibitory receptor profiles offer a biologically coherent lens through which to examine the central paradox of checkpoint blockade: the same interventions that liberate antitumor immunity can simultaneously erode self-tolerance. Their principal strength relative to conventional biomarkers lies in their closer proximity to mechanism. These receptors register the immune system’s recent antigenic history, its present regulatory constraints, and, to a meaningful extent, its latent capacity for either productive reinvigoration or injurious autoreactivity. They do not, however, convey information in a uniform language. The functional significance of PD-1, CTLA-4, LAG-3, TIM-3, TIGIT, VISTA, or NKG2A is shaped by cell lineage, differentiation stage, concurrent activating signals, and anatomical location.

Three conclusions follow. First, receptor abundance considered in isolation is rarely sufficient for clinical decision-making. More informative are composite cellular states that distinguish precursor from terminal dysfunction and that incorporate spatial organization within the tumor microenvironment. Second, toxicity prediction should be treated as a primary rather than secondary objective. Immune homeostasis, host susceptibility, and tissue-specific tolerance mechanisms must be incorporated into biomarker models from the outset. Third, the most clinically useful architecture is integrated and longitudinal. Baseline tissue data, serial blood monitoring, routine laboratory variables, autoantibody profiles, and host-level features should be synthesized within regimen-specific models that jointly estimate the probabilities of efficacy and harm.

When these conditions are met, inhibitory receptor profiling can advance beyond correlative description to become a practical instrument for patient selection, regimen tailoring, and ongoing surveillance. The field has now reached sufficient maturity to shift emphasis from descriptive checkpoint biology toward the definition of clinically interpretable therapeutic windows. This transition, more than the identification of any single marker, will ultimately determine how far precision immunotherapy can advance in routine practice.
